# Altered gut microbiota is associated with the formation of occult hepatitis B virus infection

**DOI:** 10.1128/spectrum.00239-24

**Published:** 2024-05-24

**Authors:** Bochao Liu, Hualong Yang, Qiao Liao, Min Wang, Jieting Huang, Ru Xu, Zhengang Shan, Huishan Zhong, Tingting Li, Chengyao Li, Yongshui Fu, Xia Rong

**Affiliations:** 1 Institute of Blood Transfusion and Hematology, Guangzhou Blood Center, Guangzhou Medical University, Guangzhou, Guangdong, China; 2 The Key Medical Laboratory of Guangzhou, Guangzhou, Guangdong, China; 3 Nanfang Hospital, Southern Medical University, Guangzhou, China; 4 Department of Transfusion Medicine, School of Laboratory Medicine and Biotechnology, Southern Medical University, Guangzhou, China; 5 Department of Blood Transfusion, Guangzhou First People′s Hospital, Guangzhou, Guangdong, China; University of Manitoba, Winnipeg, Canada

**Keywords:** occult hepatitis B virus infection, gut microbiota, 16S sequencing, *Subdoligranulum*, *Faecalibacterium*

## Abstract

Hepatitis B virus (HBV), a common blood transmission pathogen worldwide, can lead to viral hepatitis, cirrhosis, liver cancer, and other liver diseases. In particular, occult hepatitis B virus infection (OBI) may be caused by an immune response leading to suppressed virus replication. Gut microbiota can change the immunity status of the human body and, therefore, affect the replication of HBV. Thus, to identify whether there are differences in gut microbiota between HBV carriers and OBI carriers, we collected fecal samples from 18 HBV carriers, 24 OBI blood donors, and also 20 healthy blood donors as negative control. After 16S sequencing, we found that the abundance of *Faecalibacterium* was significantly reduced in samples from OBI blood donors compared with those from healthy blood donors. Compared with samples from HBV carriers, the samples from OBI blood donors had a significantly increased abundance of *Subdoligranulum*, which might stimulate immune activation, thus inhibiting HBV replication and contributing to the formation of occult infection. Our findings revealed the potential role of gut microbiota in the formation of OBI and further provided a novel strategy for the treatment of HBV infection.

**IMPORTANCE:**

Occult hepatitis B virus infection (OBI) is a special form of hepatitis B virus infection with hepatitis B surface antigen (HBsAg) positive and hepatitis B virus (HBV) DNA negative. Gut microbiota may contribute to the immune response leading to suppressed virus replication and, thus, participates in the development of OBI. The study on gut microbiota of OBI blood donors provides novel data considerably advancing our understanding of the immune mechanism for the determination of occult hepatitis B virus infection, which is helpful for improving the strategy of the treatment of HBV infection.

## INTRODUCTION

Hepatitis B virus (HBV) is a common blood transmission pathogen worldwide and can lead to viral hepatitis, cirrhosis, liver cancer, and other liver diseases (1). There are approximately 300 million HBV carriers worldwide, including over 100 million in China, indicating a highly endemic area of hepatitis B virus infection (2). Occult hepatitis B virus infection (OBI) is a special form of hepatitis B virus infection, which is defined as the negative detection of hepatitis B surface antigen (HBsAg) with HBV DNA-positive blood or liver tissue (3). OBI blood donors can transmit HBV through blood transfusion, leading to the development of typical hepatitis B in the recipients (4). When patients with OBI receive cancer chemotherapy or other immunosuppressive therapies that can suppress the immune system, HBV reactivation could occur (5, 6). Therefore, some studies suggested that HBV strains in OBI carriers were wild-type without Pre-S/S gene mutations, and occult infection was caused by the body’s immune response, which resulted in extremely low levels of HBV replication and expression (7).

Gut microbiota (GM) comprises a group of microbiota designated for the gastrointestinal tract, which co-evolved with the host, and plays an important role in human metabolism and immune regulation (8). Gut microbiota is important in promoting the development of the immune system, maintaining normal immune function, and cooperating against the invasion of pathogens (9). The gut and the liver have mutual interactions between both organs and microbiota. The sum of these interactions is conceptualized as the gut–liver axis (10). Thus, the gut microbiota is closely related to liver diseases, including non-infectious and infectious liver diseases such as HBV-induced chronic liver disease (11–14). Some studies have shown that composition of gut microbiota appears to vary significantly between healthy people and HBV patients at different stages, which indicated that gut microbiota might provide novel therapeutic options in HBV patients (15, 16). The composition of gut microbiota may change the immunity status of human bodies, thus affecting the replication of HBV. OBI may be due to an immune response leading to suppressed virus replication. We speculated whether gut microbiota contributes to the immune response and, thus, participates in the development of OBI. To test this hypothesis, we collected 18 fecal samples from HBV carriers as positive controls, 20 fecal samples from healthy blood donors as negative controls, and 24 fecal samples from OBI blood donors. By using 16S sequencing, we evaluated whether the differential gut microbiota composition in fecal samples from OBI blood donors might induce an immune response and, thus, is associated with the formation of OBI, which may provide a novel strategy for the treatment of HBV infection.

## MATERIALS AND METHODS

### Study cohort

This project was approved by the Ethics Committee of the Guangzhou Blood Center, and the study protocol conformed to the ethical guidelines of the Declaration of Helsinki. Informed consent was obtained from all participants before their enrollment.

A total of 20 healthy Chinese blood donors were enrolled at the Guangzhou Blood Center from December 2021 to June 2022. The exclusion criteria were as follows: (i) gastrointestinal diseases; (ii) autoimmune disease; (iii) history of antibiotics within 6 months; (iv) history of alcohol abuse; (v) history of gastrointestinal surgery; or (vi) diabetes, hypertension, or metabolic syndrome.

A total of 42 age- and BMI-matched HBV carriers (HBsAg+/DNA+, *n* = 18) or OBI blood donors (HBsAg−/DNA+, *n* = 24) were enrolled at the Guangzhou Blood Center. The criteria for HBV carriers or OBI blood donors were consistent with a previous study (17). The exclusion criteria were (i) gastrointestinal diseases; (ii) liver diseases such as acute hepatitis, liver fibrosis, cirrhosis, and liver cancer; (iii) autoimmune disease; (iv) history of antibiotics within 6 months; (v) history of alcohol abuse; (vi) history of gastrointestinal surgery; or (vii) diabetes, hypertension, or metabolic syndrome.

All blood donors among the three groups were chosen from the same district (Guangzhou) with similar dietary habits (all from Guangfu people). Before the fecal samples were collected, all blood donors among the three groups were inquired about their detailed dietary habits, and only the blood donors with a light diet (no spicy, pickled, or fried food) were included.

### Sample collection and preparation

Fecal samples from healthy blood donors, HBV carriers, or OBI blood donors participating in follow-up were collected. If the sample conformed to the requirements (smooth and soft stool that was sausage- or snake-shaped), it was retained for the study and immediately frozen at −80°C. The collection and storage process was completed within 30 min. DNA was extracted using the TGuide S96 Magnetic Stool DNA kit (Tiangen Biotech, Beijing, China) according to the instructions. DNA quantification and purity were assessed using a NanoDrop 2000 (Thermo Scientific, MA, USA).

### PCR amplification

The V3-V4 hypervariable region of the bacterial 16S ribosomal RNA (rRNA) gene was amplified with the forward primers 338F (5′-ACTCCTACGGGAGGCAGCA-3′) and the reverse primers 806R (5′-GGACTACHVGGGTWTCTAAT-3′). The PCR conditions were as follows: initial denaturation at 95°C for 5 min; 25 cycles of denaturation at 95°C for 30 s, annealing at 50°C for 30 s, and extension at 72°C for 40 s; and a final extension at 72°C for 7 min. The PCR products were extracted from 1.8% agarose gel and purified using an AxyPrep DNA Gel Extraction kit (Axygen Biosciences, California, USA) according to the manufacturer’s protocol.

### Illumina NovaSeq sequencing

Purified amplicons were pooled in equimolar amounts and were paired-end-sequenced on an Illumina NovaSeq 6000 platform (Illumina, San Diego, USA) according to the PE250 standard protocols by Biomarker Technology Co. Ltd. (Beijing, China). The raw gene sequencing reads were merged using FLASH version 1.2.11. The concatenated sequences were quality-filtered by Trimmomatic version 0.33, and chimeras were removed using UCHIME version 8.1. Operational taxonomic units (OTUs) with a 97% similarity cutoff were clustered using USEARCH version 10.0 and filtered with a threshold of 0.005%. The raw 16S rRNA gene sequences of 62 fecal samples were deposited in the NCBI Sequence Read Archive database with accession number PRJNA1033371.

### Extracellular cytokine measurement

Peripheral blood mononuclear cells (PBMCs, 1 × 10^6^ per well) were stimulated with HBV core peptides, which included nine HLA-I and eight HLA-II restrictive core epitope peptides (Table S1) (18–20) and were prepared in a pool with 5 µg/mL of each peptide. After 5 days of incubation at 37°C in 5% CO_2_, the cytokines IFN-γ and IL-17A in the supernatants of cell cultures were quantified by the enzyme-linked immuno sorbent assay (ELISA) kit (Dakewe, Beijing, China).

### ELISpot assay

Triplicates of 2.5 × 10^5^ PBMCs per well were inoculated in 96-well plates pre-coated with anti-IFN-γ or anti-IL-17A monoclonal antibody (Dakewe, Beijing, China) and incubated for 24 h with the pooled HBV core peptides (5 µg/mL each). ELISpot was performed according to the manufacturer’s protocol.

### Statistical analysis

SPSS 21.0 (SPSS, Chicago, USA) was used for all statistical analyses. Categorical variables were analyzed by chi-squared tests. Comparisons between two groups were analyzed using the unpaired *t*-test or Mann–Whitney *U* test, while comparisons between three groups were analyzed by one-way ANOVA or the Kruskal–Wallis test. A *P*-value <0.05 was considered statistically significant.

## RESULTS

### Participant characteristics

In total, 18 HBV carriers, 24 OBI blood donors, and 20 healthy blood donors as healthy controls were enrolled. Their clinical characteristics can be found in Table 1. All participants were male, and there was no difference in body mass index (BMI), age, the proportion of drinkers or smokers, and also the count of platelets (PLTs), white blood cells (WBCs), lymphocytes, neutrophils, and monocytes. There was no difference in aspartate transaminase (AST) among the three groups while the HBV carriers had the highest alanine aminotransferase (ALT) and showed differences from the other two groups, though the ALT values of all three groups were within the normal range. The liver parameters were tested using FibroScan, and there was no difference in the liver stiffness measurement for HBV carriers and OBI blood donors, and both were within the normal range, indicating that there was no severe liver disease in these two groups. The mean value of HBsAg of the HBV carriers was 1.8 × 10^4^ IU/mL and was negative for the two other groups. The hepatitis B surface antibody (HBsAb) values for HBV carriers and OBI blood donors were all less than 100 IU/L, indicating that the two groups lacked protection against HBV. The hepatitis B core antibody (HBcAb) tests for HBV carriers and OBI blood donors were all positive while tests for healthy blood donors were all negative, indicating that all healthy blood donors have no previous infection with HBV. The quantitative detection of HBV DNA showed that the HBV carriers had significantly higher levels than the OBI blood donors.

**TABLE 1:** Clinical characteristics of subjects^*a*^

Clinical index	HBV carriers	Blood donors		*P*-value
		OBI	HC	
Numbers	18	24	20	–
Gender	Male	Male	Male	–
Age (year, mean ± SD)	36.1 ± 8.5	41.1 ± 4.7	36.5 ± 11.3	*P* = 0.11
BMI (kg/m^2^)	22.6 ± 4.7	23.7 ± 2.4	24 ± 3	*P* = 0.47
Current smoker, *n* (%)	4 (22.2)	4 (16.7)	4 (20)	*P* = 0.9
Current drinker, *n* (%)	0 (0)	0 (0)	0 (0)	*P* > 0.05
ALT (U/L)	29.3 ± 11.2	20.5 ± 8.5	19.1 ± 12.2	*P* < 0.01
AST (U/L)	30.3 ± 17.8	22.9 ± 5.7	27.2 ± 11.9	*P* = 0.15
PLT (10^9^/L）	242 ± 54	267 ± 58	287 ± 60	*P* = 0.06
WBC (10^9^/L）	6.2 ± 1.4	6.6 ± 1.2	7.2 ± 2.2	*P* = 0.21
Lymphocyte (10^9^/L）	2.1 ± 0.6	2.2 ± 0.8	2.2 ± 0.6	*P* = 0.8
Neutrophil (10^9^/L）	3.4 ± 0.8	3.7 ± 0.6	4.1 ± 1.6	*P* = 0.17
Monocyte (10^9^/L）	0.41 ± 0.11	0.39 ± 0.08	0.47 ± 0.24	*P* = 0.19
LSM (kPa)	5.2 ± 0.8	5 ± 0.9	Not detected	*P* = 0.55
HBsAg (IU/mL)	1.8 × 10^4^	Negative	Negative	*P* < 0.05
HBsAb (IU/L)	0～10	0～45	13～1,000	*P* < 0.01
HBcAb+ (%)	18 (100)	24 (100)	0 (0)	*P* < 0.05
HBV DNA (IU/mL)	1.09 × 10^2^～2.33 × 10^9^	3.4～46	Negative	*P* < 0.01

^*a*^ LSM, liver stiffness measurement. “–” means there is no P value.

**Fig 1:**
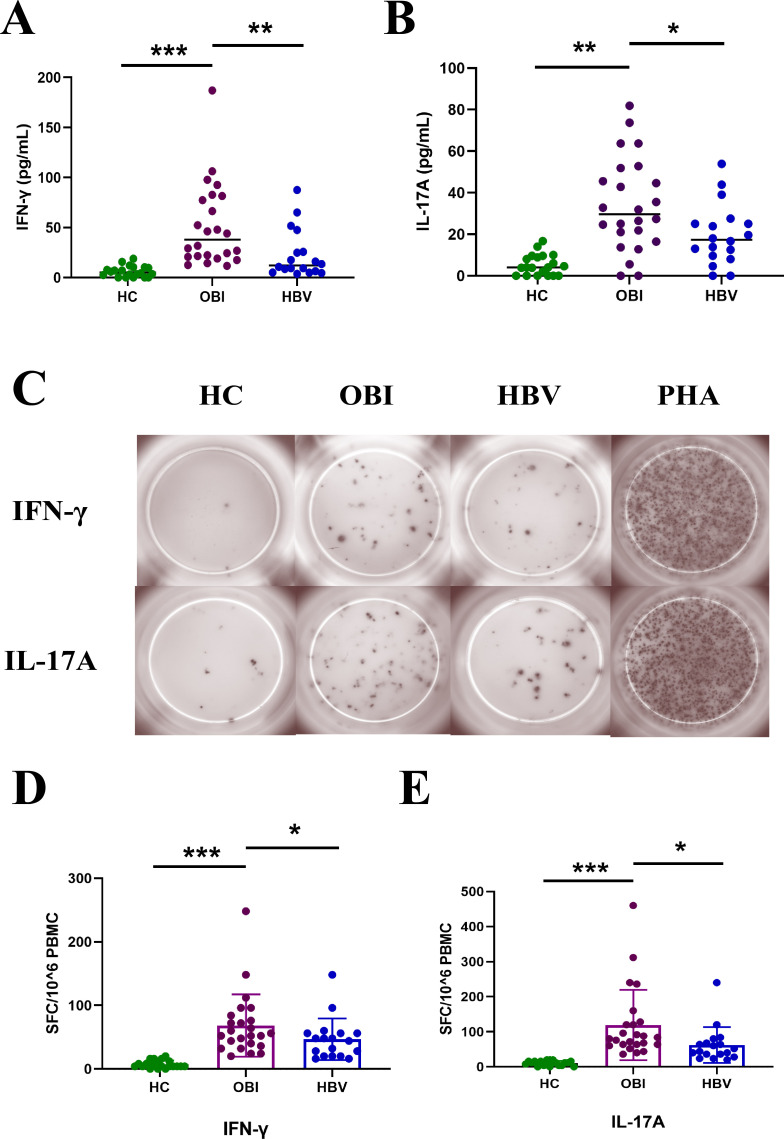
Comparison of the immune status of the healthy blood donors, HBV carriers, and OBI blood donors. Extracellular IFN-γ (**A**) and IL-17A (**B**) concentrations in the culture supernatants of PBMCs stimulated by HBV core peptides. (**C**) Representative specific and IL-17A secreting T-cell response of PBMCs by ELISpot after stimulation with HBV core peptides. The level of IFN-γ (**D**) or IL-17A (**E**) secreting T-cell response from individual donors is presented as spot-forming cells per 1 × 10^6^ PBMCs. ^*^P < 0.05, ^**^P < 0.01, and ^***^P < 0.001.

To investigate the immune status of these three groups, the secretion of cytokines IFN-γ and IL-17A in culture supernatants of PBMCs was quantified after stimulation with HBV core peptides by ELISA (Fig. 1A and B). The results showed that the level of IFN-γ and IL-17A secreted by the OBI group was higher than that obtained in HC or HBV carriers. The specific IFN-γ and IL-17A secretion T-cell response among the three groups were also found significantly different by ELISpot after stimulation with HBV core peptides, which showed a higher IFN-γ and IL-17A secreting T-cell response in the OBI group compared with HC or HBV carriers (Fig. 1C through E).

### Diversity and taxonomic distribution of the three groups

**Fig 2:**
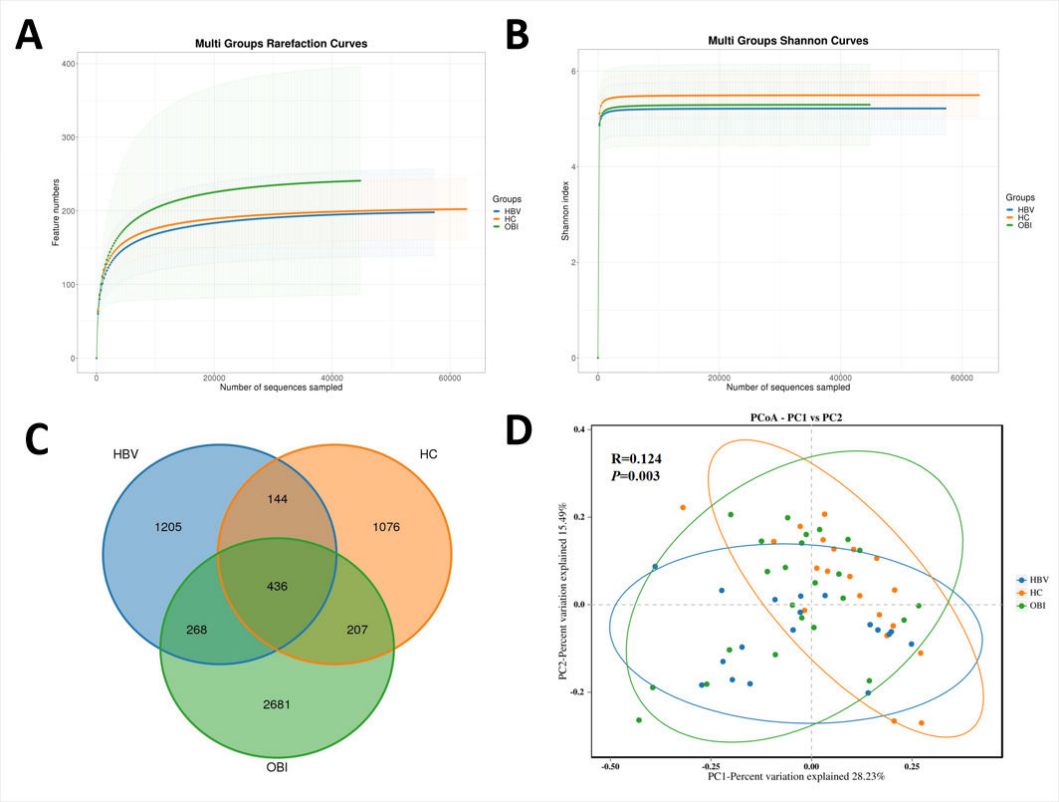
Comparison of the structures of the gut microbiota of the healthy blood donors, HBV carriers, and OBI blood donors. Rarefaction curves (**A**) and Shannon index (**B**) were used to estimate the richness of the gut microbiota of the three groups. (**C**) Common and unique OTUs among the three groups were visualized by Venn diagram. Each circle represented one group. The overlaps indicated common OTUs among groups, and the non-overlapped area indicated unique OTUs in each group. (**D**) Principal coordinate analysis (β-diversity) of the three groups. Each point in the graph represents a fecal sample, and different colors represent different groups. The elliptical circle represents a 95% confidence range, and the distance between points indicates the degree of difference. The horizontal and vertical axes represent the first and second principal components, respectively, while the percentage represents the contribution to the sample difference.

There were no significant differences in the α-diversity indexes, including Chao1, ACE, Shannon, and Simpson indexes, among these three groups (Table 2). Although the ACE and Chao1 index scores of the OBI group were higher than those of the other two groups, these differences did not reach the significance level. The structures of the gut microbiota were analyzed, which indicated that the sequencing data were adequate to present most species in the samples (Fig. 2A and B) and also showed differences between samples among these three groups (Fig. 2C and D).

**TABLE 2:** α-Diversity index analysis

Group	ACE	Chao1	Simpson	Shannon
HC	204.28 ± 40.31	203.3 ± 40.21	0.95 ± 0.022	5.496 ± 0.438
OBI	244.51 ± 151.95	243.87 ± 151.99	0.93 ± 0.072	5.297 ± 0.834
HBV	199.89 ± 57.87	199.11 ± 57.61	0.935 ± 0.034	5.218 ± 0.538

**Fig 3:**
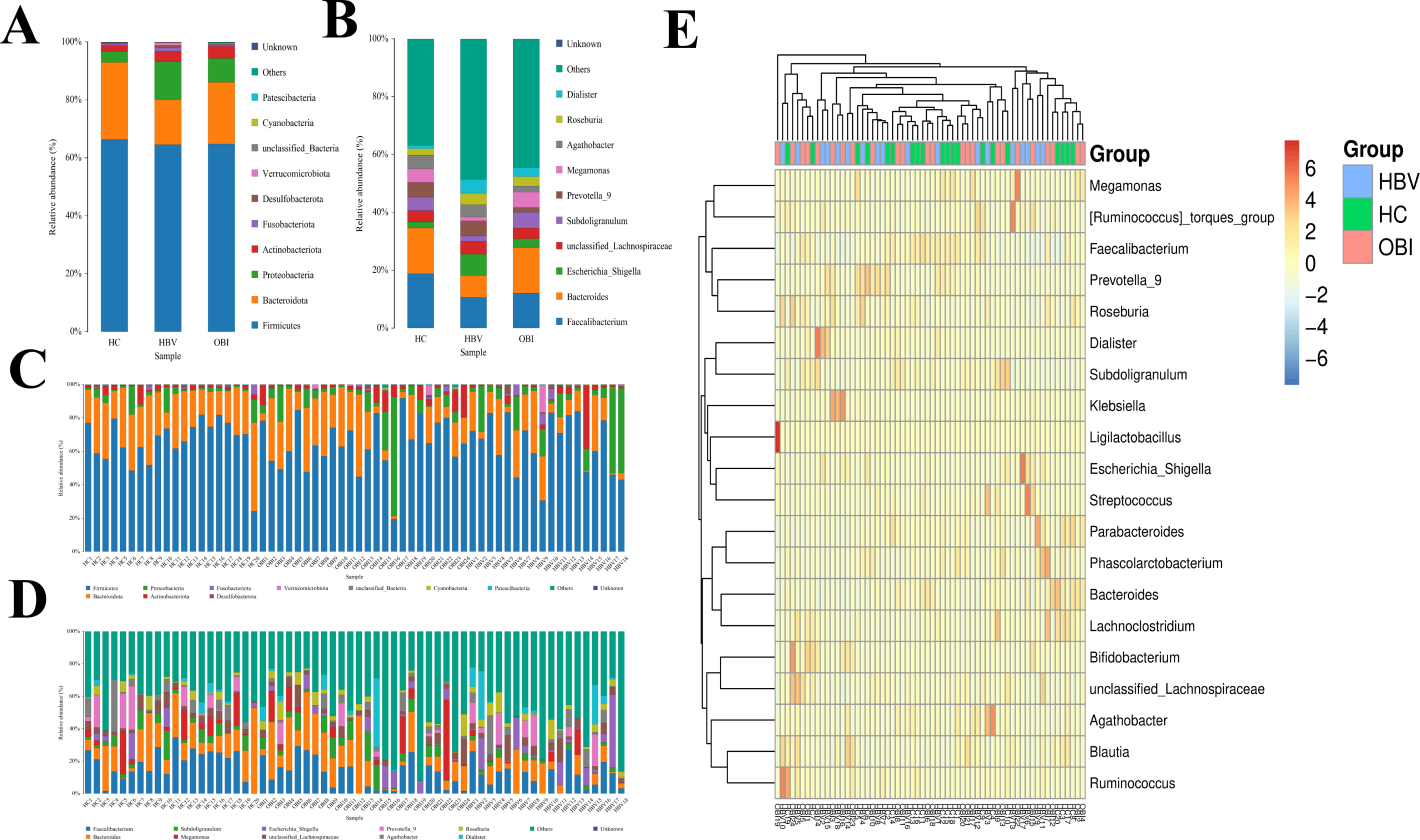
The changes in the gut microbiota of the HBV and OBI groups compared to healthy controls at the phylum (**A**) and genus (**B**) levels. The phylum (**C**) and genus (**D**) composition of gut microbiota of each sample in HC, OBI, or HBV group. The column showed only the top 10 phyla or genera with abundance levels. (**E**) Heatmap indicating the genus-level changes in HC, OBI, or HBV group.

At the phylum level, the OTUs were assigned to nine known phyla. The predominant phylum was *Firmicutes*, followed by *Bacteroidota*, *Proteobacteriota*, and *Actinobacteriota* (Fig. 3A). At the genus level, the OTUs were assigned to 10 known genera, including *Faecalibacterium*, *Bacteroides*, *Megamonas*, and *Subdoligranulum* (Fig. 3B). The phylum and genus composition of gut microbiota of each sample in the HC, OBI, and HBV groups is shown in Fig. 3C and D. The heatmap showed the correlations between the participants and the abundance levels of selected genera that were represented in the microbiota samples (Fig. 3E).

### Altered gut microbiota between HBV carriers and OBI blood donors

**Fig 4:**
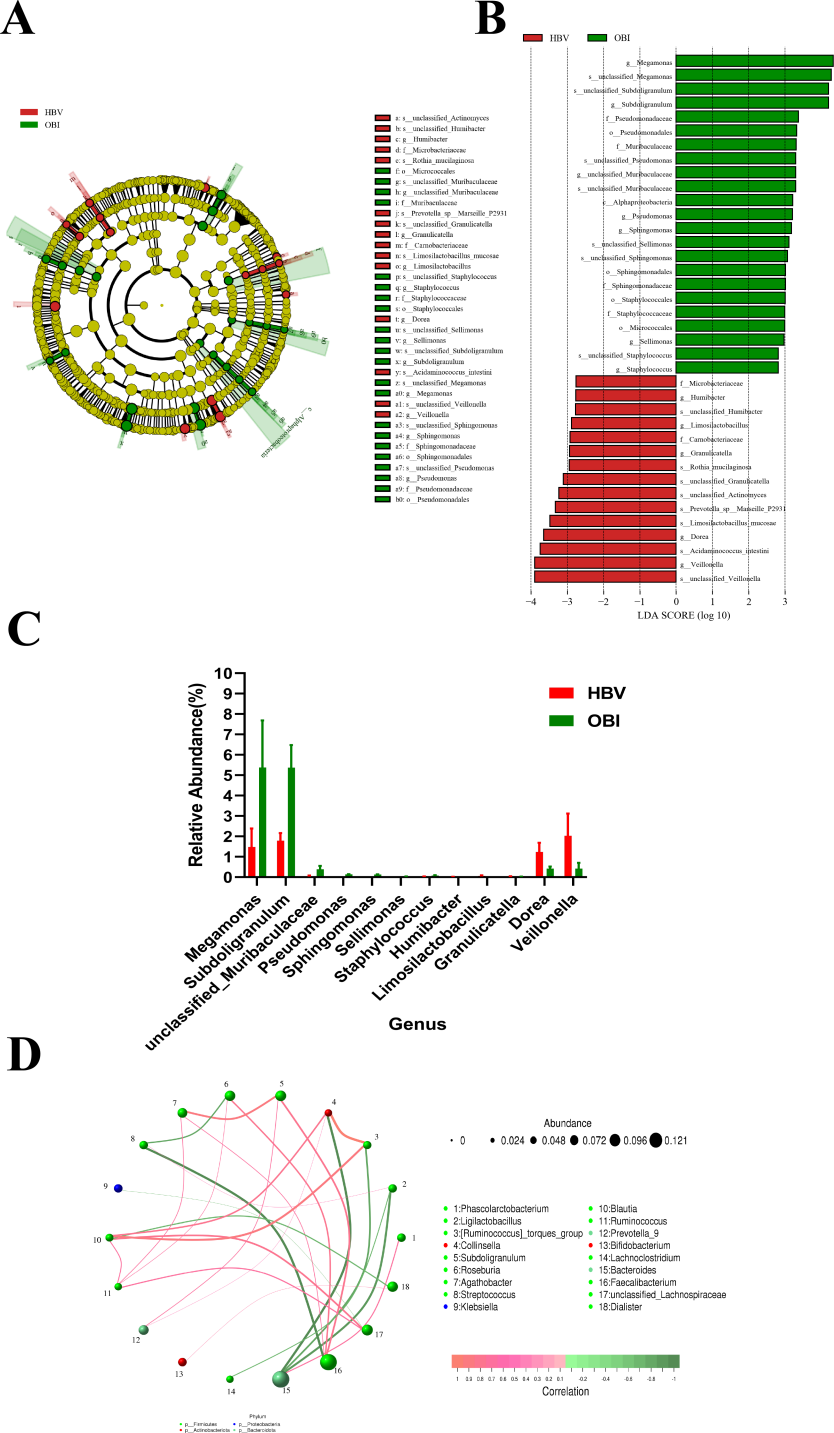
Characteristics of altered gut microbiota between HBV carriers and OBI blood donors. The differences in gut microbiota are shown through LEfSe cladogram (**A**) and histogram (**B**) (LDA = 2). (**C**) Among the differential bacterial compositions, the abundances of genera between HBV carriers and OBI blood donors. (**D**) Correlation network analysis of gut microbiome at the level of genus between HBV carriers and OBI blood donors. The circle represents the different genera, and the size of circles represents the average abundance of the genera. The line represents the correlation between two genera, and the thickness of lines represents the strength of correlation. The color of lines represents positive (red) or negative (green) correlation.

The gut microbiota of HBV carriers and OBI blood donors were analyzed by line discriminant analysis effect size (LEfSe), and the results are shown in Fig. 4A and B. Among the differential bacterial compositions, the abundances of seven genera were relatively high in the OBI group, while five genera were relatively high in the HBV group (Fig. 4C). The LDA line discriminant analysis (LDA) values and abundances of *Megamonas* and *Subdoligranulum* were highest among these differential genera. The abundance of *Subdoligranulum* was 1.79% in HBV carriers, which was lower than that of the OBI group (5.37%) as analyzed by an *F*-test (*P* < 0.05). The abundance of *Subdoligranulum* was at an intermediate level in the HC group and did not have a statistical difference compared with the other two groups (data not shown). By constructing the correlation network and analyzing the interactions at the genus level, it was found that the *Subdoligranulum* displayed positive associations with *Agathobacter*, *Prevotella*, and *Faecalibacterium* (Fig. 4D).

### Altered gut microbiota between healthy controls and OBI blood donors

**Fig 5:**
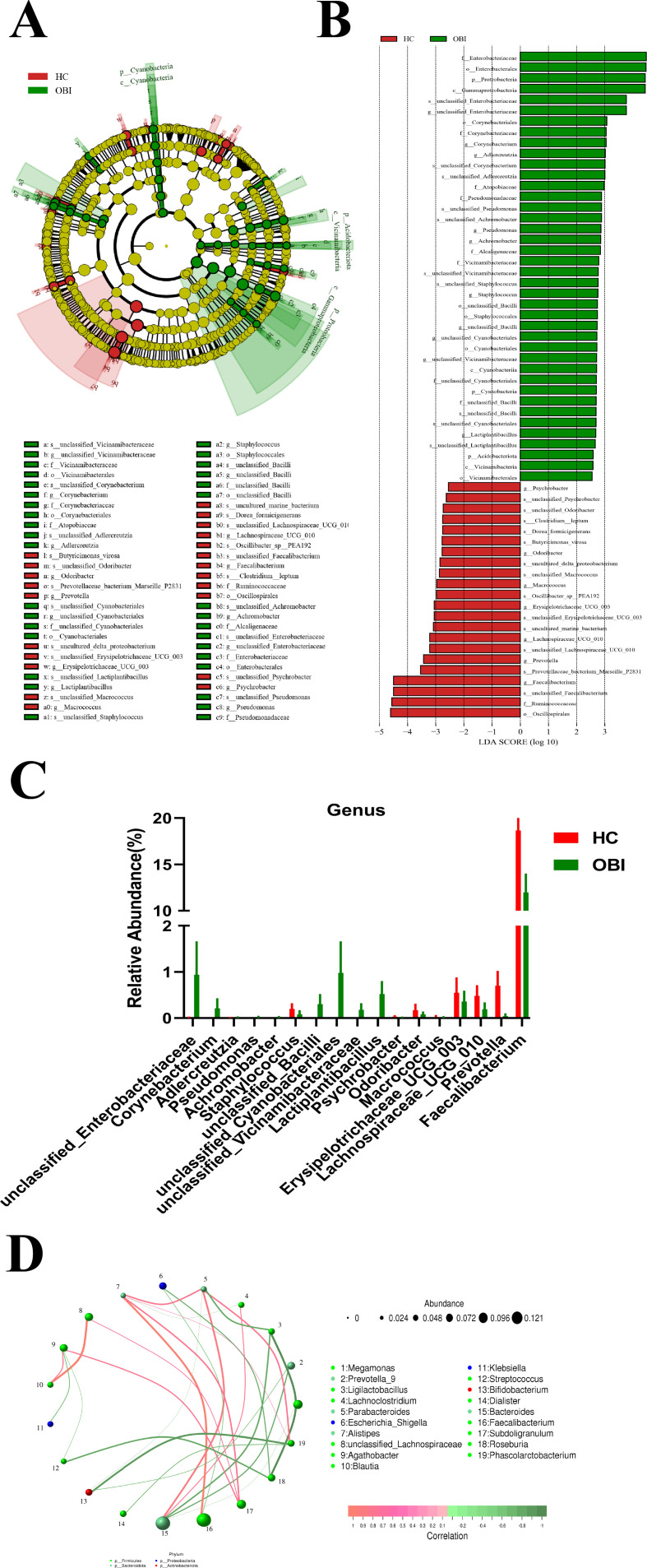
Characteristics of altered gut microbiota between healthy controls and OBI blood donors. The differences in gut microbiota are shown through LEfSe cladogram (**A**) and histogram (**B**) (LDA = 2). (**C**) Among the differential bacterial compositions, the abundances of genera between healthy controls and OBI blood donors. (**D**) Correlation network analysis of gut microbiome at the level of genus between healthy controls and OBI blood donors. The circle represents the different genera, and the size of circles represents the average abundance of the genera. The line represents the correlation between two genera, and the thickness of lines represents the strength of correlation. The color of lines represents positive (red) or negative (green) correlation.

The results of the gut microbiota comparison between healthy controls and OBI blood donors are shown in Fig. 5A and B. Among the differential bacterial composition, the abundances of 10 genera were relatively high in the OBI group, while seven genera were relatively high in the HC group (Fig. 5C). The LDA value and abundance of *Faecalibacterium* were highest among these differential genera. The abundance of *Faecalibacterium* in the HC group was also higher compared with that of HBV carriers (Fig. S1). By constructing the correlation network and analyzing the interactions at the genus level, *Faecalibacterium* was found to display positive associations with *Alistipes* but negative associations with *Escherichia* (Fig. 5D).

### KEGG metabolic pathway analysis shows the proportion of different functions among the three groups

**Fig 6:**
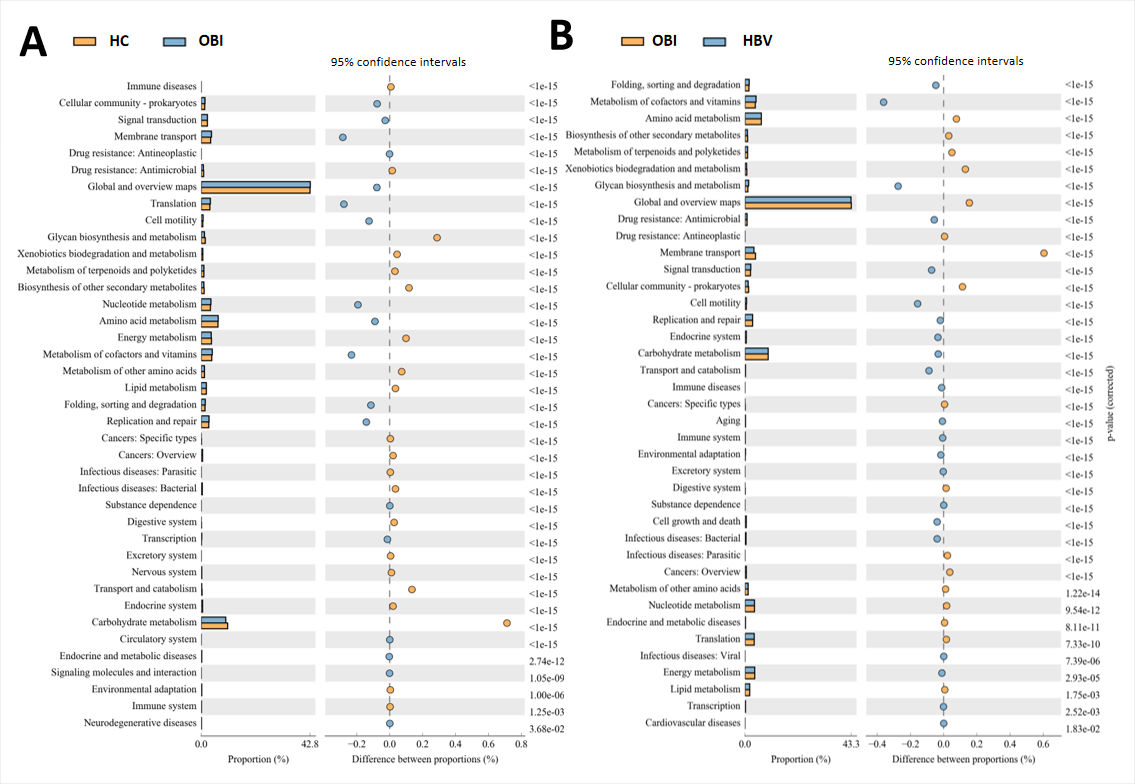
KEGG pathway analysis of gut microbiota of the OBI blood donors compared to healthy controls (**A**) or HBV carriers (**B**).

**Fig 7:**
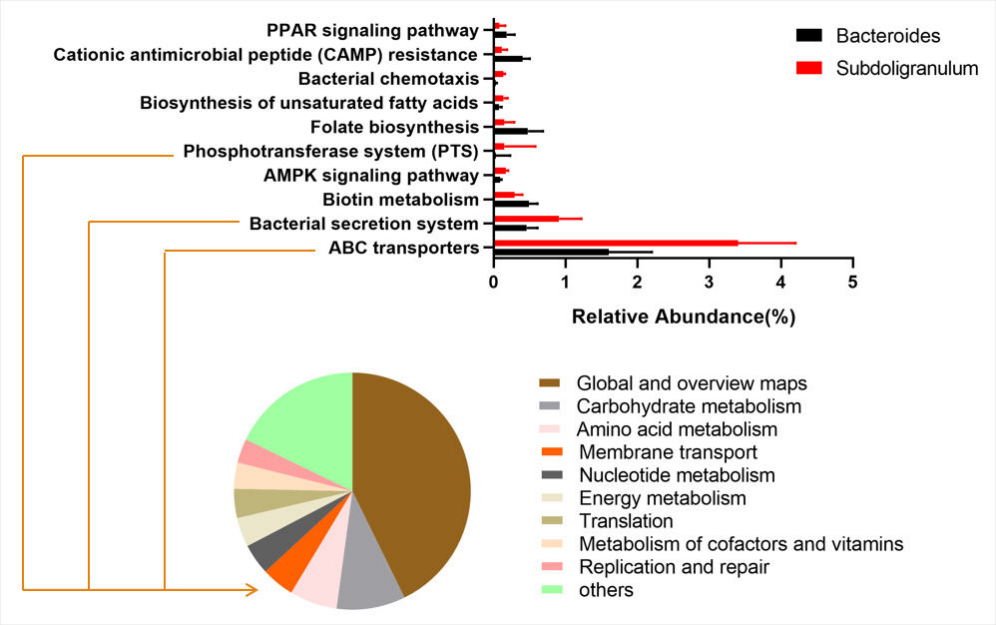
The KEGG-based functional gene prediction of *Subdoligranulum.* The percentages of relative abundance of the top 10 functional pathways with the greatest changes compared with *Bacteroides* in which three of them belonged to class of membrane transport in KEGG-based functional gene prediction.

Kyoto Encyclopedia of Genes and Genomes (KEGG) is a utility database resource for understanding advanced functions and biological systems. By analyzing the composition of the KEGG metabolic pathways, the differences and changes in the metabolic pathways of functional genes in the microbial communities in samples of the three groups were determined, as shown in Fig. 5. Using KEGG-based function prediction, the metabolic pathway with the greatest difference in proportion between the OBI and HC groups was carbohydrate metabolism (Fig. 6A). When compared with HBV carriers, the samples of OBI blood donors had the maximum upregulation in the metabolic pathway of membrane transport (Fig. 6B). While compared with *Bacteroides*, which was one of the most common genera in human gut microbiota, 3 of the top 10 functional pathways with the greatest changes of relative abundance in *Subdoligranulum* also belonged to the class of membrane transport (Fig. 7).

## DISCUSSION

GM comprises a group of microbiota that co-evolved with the host and plays an important role in human nutrition metabolism and immune regulation. Metabolites of gut microbiota may affect the immune response of the human body by interacting with different participants, such as intestinal lymphocytes (21). The gut–liver axis refers to the bidirectional relationship between the gut and the liver, resulting from the integration of signals generated by microbiota and other environmental factors, which is established by the portal vein and enables transport to the gut and liver (22). Thus, changes in metabolites produced by gut microorganisms can contribute to liver diseases. In recent years, there have been increasing researches on the relationship between GM and HBV-induced chronic liver diseases (16, 23–29). A recent study has revealed the differences in the diversity and the composition of gut microbiota between healthy people and HBV patients at different stages. As HBV infection remains difficult to cure despite the development of antiviral therapies, the results indicate that the understanding of gut microbiota may help develop effective treatment strategies for HBV patients (16).

Liver damage in patients with hepatitis B causes edema of the intestinal wall and affects intestinal peristalsis, thus causing the destruction of the intestinal mucosal barrier. The disruption of the intestinal mucosal barrier function can lead to the translocation of bacteria and their metabolites and changes in the GM composition (a decrease in beneficial bacteria and excessive growth of pathogenic bacteria) (30). Furthermore, the damage to the intestinal mucosal barrier and the change of GM may cause an increase in harmful substances and inflammatory factors in patients with hepatitis B, further aggravating liver injury (15). The host immune response is considered to be one of the main factors in the occurrence and development of hepatitis B. A recent study has shown that the damage to hepatocytes in patients with hepatitis B comes not only from the immune response caused by HBV but also from the innate immune response induced by pathogen-associated molecular patterns due to a GM imbalance (31). Gut microbiota was also shown to be closely related to hepatitis B virus infection. In the study of a mouse model, it was found that when antibiotics were used to deplete the gut microbiota, HBV infection in mice became chronic, suggesting that gut microbiota might have suppressed HBV replication (32). We speculated whether the formation of OBI was due to the difference in the composition of gut microbiota between OBI carriers and patients with overt HBV infection, such as a higher proportion of probiotics that could help activate an immune response and, thus, inhibit HBV replication. To test this hypothesis, we collected 18 fecal samples from HBV carriers as positive controls, 20 fecal samples from ordinary blood donors as negative controls, and 24 fecal samples from OBI blood donors. By using 16S sequencing, we analyzed the differential gut microbiota composition in fecal samples from OBI blood donors that may induce an immune response and were, therefore, associated with the formation of OBI, which may provide a novel strategy for the treatment of HBV infection.

In the OBI group, the abundance of *Subdoligranulum* was significantly increased compared to that of HBV carriers (Fig. 3C). A recent study has shown that the *Subdoligranulum* played an immune regulatory role in autoimmune diseases. For individuals at risk for rheumatoid arthritis, compared to controls, *Subdoligranulum* stimulated Th17 cell expansion and drove systemic antibody generation and immune activation (33). Interestingly, the secretion of cytokines IFN-γ and IL-17A and the IFN-γ and IL-17A secreting T-cell response of the OBI group were at a higher level compared with HC or HBV carriers, which indicated different immune status of these three groups. Considering the role of IFN-γ and IL-17A in HBV infection (34–36), the OBI group exhibited a much stronger immune response to suppress HBV replication compared with HC or HBV carriers (Fig. 1). These results indicated that the *Subdoligranulum* might play a role in stimulating immune activation, thus inhibiting HBV replication and contributing to the formation of occult infection. The abundance of *Faecalibacterium* in the HC group was significantly increased compared to the other two groups and is considered a probiotic with anti-inflammatory functions (37, 38) and, thus, might indicate the presence of chronic mild inflammation in the liver of both HBV carriers and OBI blood donors.

The correlation network analysis included a Spearman rank correlation analysis based on the abundance and changes of each genus, and data were selected with a correlation >0.1 and *P* < 0.05 to construct a correlation network. Based on the correlation network analysis, a co-existence relationship of genera was obtained, along with interaction and important pattern information, further explaining the formation mechanism of the differences between samples. By constructing the correlation network and analyzing the interactions at the genus level, it was found that *Subdoligranulum* displayed positive associations with *Agathobacter*, *Prevotella*, and *Faecalibacterium*, which might be associated with an augmented T helper type 17 (Th17)-mediated immune response (39) (Fig. 3D). Additionally, *Faecalibacterium* displayed positive associations with *Alistipes*, which is capable of producing short-chain fatty acids and reducing intestinal inflammation (40), while negative associations with *Escherichia_Shigella* are related to intestinal inflammation and infection (41) (Fig. 4D).

The KEGG-based metabolic pathway analysis and function prediction showed that the samples of healthy controls had the maximum upregulation in the metabolic pathway of carbohydrate metabolism, compared with that of OBI blood donors, which might be associated with the differences in the gut microbiota composition between the OBI and HC groups. The metabolic pathway of functional genes of the OBI group showed the highest upregulation compared to that of the HBV carriers in terms of membrane transport, which referred to the process of regulating solutes such as ions and small molecules passing through biofilms. Interestingly, as compared with *Bacteroides,* 3 of the top 10 functional pathways with the greatest changes of relative abundance in *Subdoligranulum* belonged to class of membrane transport, which also indicated that the difference in gut microbiota composition between the OBI group and HBV carriers might be associated with changes in membrane transport function.

Our study has some limitations. First, as the percentage of OBI in blood donors was 1:1,494 and most OBI blood donors were male (42), the sample size was limited and only male blood donors were included in this subject. Second, as there was no complete standardization of the food intake for the three groups, the effect of dietary differences on the gut microbiota among these three groups could not be ruled out completely. To offer in-depth research about the metabolic function of *Subdoligranulum* in HBV infection, more fecal samples from donors accepting a standardized diet should be collected and more experiments performed in future studies.

### Conclusions

In this study, we collected fecal samples from 18 HBV carriers, 20 healthy blood donors as healthy controls, and 24 OBI blood donors. Using 16S sequencing for analyzing the differences in the gut microbiota composition among these three groups, we found that compared with samples from HBV carriers, the samples from OBI blood donors had a significantly increased abundance of *Subdoligranulum*, which might stimulate immune activation and contribute to the formation of occult infection. Our findings may reveal the role of gut microbiota in the formation of OBI and further provide a novel strategy for the treatment of HBV infection.

## Data Availability

All data are available from the main article and supplemental information.

## SUPPLEMENTAL MATERIAL

Supplemental material spectrum.00239-24-s0001.doc Table S1; Fig. S1.

ASM does not own the copyrights to Supplemental Material that may be linked to, or accessed through, an article. The authors have granted ASM a non-exclusive, world-wide license to publish the Supplemental Material files. Please contact the corresponding author directly for reuse.
