# Supplementary material for: Altered gut microbiota is associated with the formation of occult hepatitis B virus infection
Source: Microbiol Spectr. 2024 May 24;12(7):e00239-24. doi: 10.1128/spectrum.00239-24 (PMC11218497; doi:10.1128/spectrum.00239-24)
Supplement: Supplemental material — Table S1; Fig. S1. [file spectrum.00239-24-s0001.doc]

**Altered Gut Microbiota is Associated with the Formation of Occult Hepatitis B Virus Infection**

Bochao Liu^1,2*^, Hualong Yang^3*^, Qiao Liao^1,2^, Min Wang^1,2^, Jieting Huang^1,2^, Ru Xu^1,2^, Zhengang Shan^1,2^, Huishan Zhong^1,2^, Tingting Li^4^, Chengyao Li^4^, Yongshui Fu^#1,2,3,5^, Xia Rong^#1,2^

^1^Institute of Clinical Blood Transfusion, Guangzhou Blood Center, Guangzhou, Guangdong, China

^2^The Key Medical Laboratory of Guangzhou, Guangzhou, Guangdong, China.

^3^Nanfang Hospital, Southern Medical University, Guangzhou, China;

^4^Department of Transfusion Medicine, School of Laboratory Medicine and Biotechnology, Southern Medical University, Guangzhou, China;

^5^Department of Blood Transfusion, Guangzhou First People′s Hospital, Guangzhou, Guangdong, China.

* These authors contributed equally to this work.

^#^ **Corresponding author:** Xia Rong or Yongshui Fu, Institute of Clinical Blood Transfusion, Guangzhou Blood Center, Guangzhou, Guangdong, China.

Email: [joyjoy@126.com](mailto:joyjoy@126.com) (X Rong); or [fuyongshui@sina.com](mailto:fuyongshui@sina.com) (Y Fu). Phone: 0086-20-83593815

| **Table S1**. HLA-I/II restricted HBV core peptides | | | |
| --- | --- | --- | --- |
| HLA class Ⅰ restricted epitopes | | HLA class Ⅱ restricted epitopes | |
| Residues | Amino acid sequence | Residues | Amino acid sequence |
| core11-27 | ASVELLSFLPSDFFPSI | core1-20 | MDIDPYKEFGASVELLSFLP |
| core18-27 | FLPSDFFPSI | core18-27 | FLPSDFFPSI |
| core19-27 | LPSDFFPSI | core28-47 | RDLLDTASALYREALESPEH |
| core88-96 | YVNVNMGLK | core50-69 | PHHTALRQAILCWGELMNLA |
| core107-115 | CLTFGRETV | core111-125 | GRETVLEYLVSFGVW |
| core115-124 | VLEYLVSFGV | core117-131 | EYLVSFGVWIRTPPA |
| core117-125 | EYLVSFGVW | core120-139 | VSFGVWIRTPPAYRPPNAPI |
| core139-148 | ILSTLPETTV | core147-156 | TVVRRRGRSP |
| core141-151 | STLPETTVVRR |  |  |


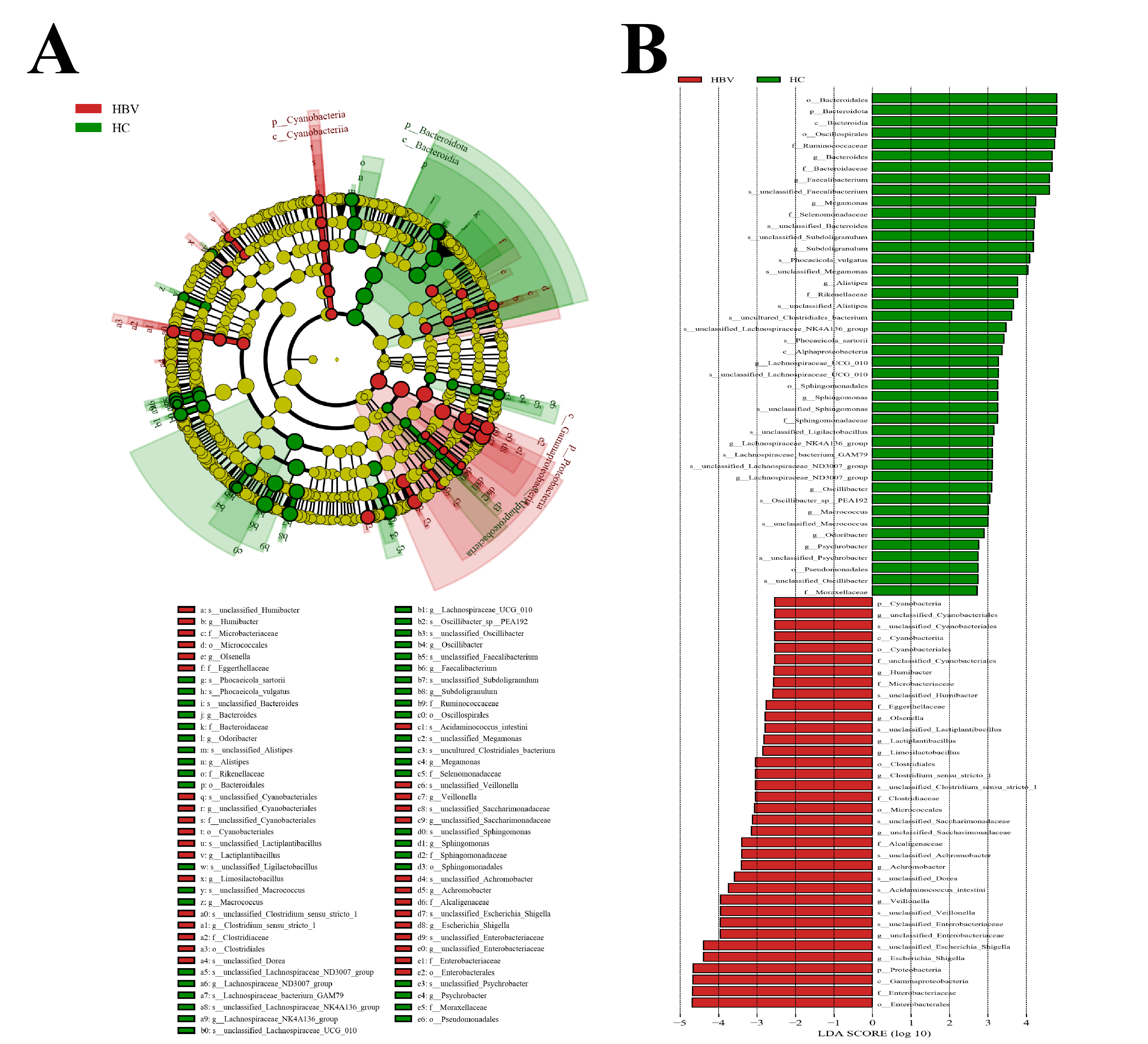


**Figure S1.** Characteristics of altered gut microbiota between healthy controls and HBV carriers. The differences in gut microbiota are shown through LEfSe cladogram (**A**) and histogram (**B**) (LDA = 2).
